# Projecting patterns of health inequalities in England up to 2040 using linked health data

**DOI:** 10.23889/ijpds.v9i5.2794

**Published:** 2024-09-18

**Authors:** Ann Raymond, Toby Watt, Laurie Rachet-Jacquet, Hannah-Rose Douglas, Anna Head, Chris Kypridemos

**Affiliations:** 1 REAL Centre, Health Foundation; 2 Department of Public Health, Policy and Systems, University of Liverpool

## Objective

The existence of wide inequalities in self-reported health across England is well-documented. Our research adds to this evidence by describing current patterns and projecting future patterns of inequality in diagnosed illness by deprivation.

## Approach

We used a microsimulation model which combines individual-level data on demographics, health and mortality from linked data for primary and secondary care with survey responses on leading modifiable risk factors and epidemiological evidence on the associations between risk factors and chronic illness.

We used the Cambridge Multimorbidity Score to measure multimorbidity. This assigns a weight to 20 common long-term conditions based on individuals’ healthcare use and their likelihood of death. We further focus on “major illness” which corresponds to a score greater than 1.5.

## Results

We project that health inequalities are not projected to improve between 2019 and 2040. In 2040, we project the difference in the average time spent without major illness between the most and least deprived 10% of areas in England to be 10.7 years. This is largely unchanged from 10.4 years in 2019.

In 2040, we project there will be more working age adults living with major illness in the most deprived areas, more than double the rate in the least deprived areas (15.2% versus 6.8%). These rates remain largely unchanged from 2019 at 14.6% and 6.3% respectively.

## Conclusions and Implications

On current trends, health inequalities are projected to persist into the future. This has significant implications not just for population health but for labour supply and wider economic growth.

